# Role of Antimicrobial Susceptibility Testing before First-Line Treatment Containing Clarithromycin for *Helicobacter pylori* Eradication in the Clinical Setting

**DOI:** 10.3390/antibiotics10020214

**Published:** 2021-02-21

**Authors:** Seokin Kang, Yuri Kim, Ji Yong Ahn, Hwoon-Yong Jung, Nayoung Kim, Hee Kyong Na, Jeong Hoon Lee, Kee Wook Jung, Do Hoon Kim, Kee Don Choi, Ho June Song, Gin Hyug Lee

**Affiliations:** 1 Asan Medical Center, Department of Gastroenterology, University of Ulsan College of Medicine, Seoul 05505, Korea; SeokinKang.MD@gmail.com (S.K.); arcoiris0209@gmail.com (Y.K.); hkna77@naver.com (H.K.N.); jhlee.gi@amc.seoul.kr (J.H.L.); jung.keewook30@gmail.com (K.W.J.); dohoon.md@gmail.com (D.H.K.); keedon@amc.seoul.kr (K.D.C.); hjsong@amc.seoul.kr (H.J.S.); jhlee409@amc.seoul.kr (G.H.L.); 2 Asan Medical Center, Department of Clinical Epidemiology and Biostatistics, University of Ulsan College of Medicine, Seoul 05505, Korea; nyny0803@amc.seoul.kr

**Keywords:** *Helicobacter pylori*, *Helicobacter* infection, clarithromycin, microbial sensitivity tests, microbial drug resistance

## Abstract

Background: Checking *Helicobacter pylori* susceptibility tests in the clinical setting before first-line treatment is considered difficult. We compared susceptibility-guided therapy (SGT) with empirical therapy (ET) as a first-line treatment containing clarithromycin and investigated the eradication rate using antimicrobial susceptibility testing (AST). Methods: 257 patients with *H. pylori* infection, with AST, performed before the eradication of clarithromycin-containing regimens were enrolled and divided into two groups: the SGT and ET groups. Results: Eradication rates in the SGT and ET groups were 85.4% and 58.4% (*P* < 0.01), respectively. In triple therapy (TT), eradication rates of the SGT and ET groups were 85.1% and 56.6% (*P* < 0.01), respectively. In sequential therapy (SET), eradication rates of the SGT and ET groups were 86.2% and 65.6% (*P* = 0.06), respectively. According to AST, TT had an eradication rate of 84.6% with strains susceptible to clarithromycin and amoxicillin and 11.1% with strains resistant to both. SET had an eradication rate of 89.5% with strains susceptible to clarithromycin, amoxicillin, and metronidazole, whereas it was 0% with strains resistant to clarithromycin and metronidazole. Conclusions: SGT as first-line treatment improved eradication rates of TT and SET by 28.5 (*P* < 0.01) and 20.6 (*P* = 0.06) percent points, respectively, compared with ET.

## 1. Introduction

*Helicobacter pylori (H. pylori)* is one of the most common pathogens causing chronic infection in humans and infecting approximately 4.4 billion people globally [1]. In Korea, the prevalence rate of *H. pylori* infection is 51.0%, according to a Korean nationwide study [2]. *H. pylori* can survive in the acidic environment of the stomach and is a major risk factor for gastritis, peptic ulcer disease, and gastric cancer. It has also been accepted as a risk factor for gastric mucosa-associated lymphoid tissue lymphoma and extragastrointestinal diseases, including immunologic impairment and cardiovascular diseases [3,4,5,6]. Eradication of *H. pylori* may decrease the incidence rate of such diseases and is important in promoting public health. *H. pylori* is in the replicative phase at neutral pH (6–7), which is susceptible to antibiotics and is in the coccoid form at acid pH (3–6), which is resistant to antibiotics [7,8,9,10]. Thus, proton pump inhibitors play an important role in eradicating *H. pylori* by increasing intragastric pH. Clarithromycin is bacteriostatic and binds to the 50S ribosomal subunit to block protein synthesis of *H. pylori*. Metronidazole is bactericidal and enters membranes, leading to the production of toxic metabolites, such as free radicals within *H. pylori*. Amoxicillin is bactericidal and inhibits the synthesis of *H. pylori* cell walls.

The Korean Society of Gastroenterology guideline recommends clarithromycin triple therapy (TT) regimen as a first-line treatment for *H. pylori* infection [11] because clarithromycin is the core antibiotic for eradication *H. pylori* [12,13]. However, the efficacy of clarithromycin-containing regimens has decreased due to an increase in clarithromycin resistance rates [14,15,16,17]. The American College of Gastroenterology guideline suggests not only clarithromycin TT regimen as a first-line treatment but also other numerous regimens, including bismuth quadruple therapy, concomitant therapy, sequential therapy (SET), hybrid therapy, levofloxacin triple therapy, and fluoroquinolone sequential therapy regimens [6]. Maastricht guidance document recommends performing clarithromycin susceptibility testing before the initiation of clarithromycin-based treatment where clarithromycin resistance is more than 15% [18].

It is difficult to check the results of *H. pylori* susceptibility testing before the initiation of eradication therapy in the clinical setting. First, failure of *H. pylori* culture is not uncommon [19,20]. Second, after *H. pylori* are cultured, it usually takes 10 days to even two weeks to obtain the results of susceptibility testing [21]. In case a patient visits the clinic before the results are reported, a physician cannot start eradication therapy based on antimicrobial susceptibility and should provide empirical therapy.

In this study, by retrospectively analyzing patient medical records, we compared susceptibility-guided therapy with empirical therapy as a first-line treatment containing clarithromycin for *H. pylori* eradication and investigated the eradication rate according to antimicrobial susceptibility testing.

## 2. Materials and Methods

### 2.1. Patients

**Figure 1:**
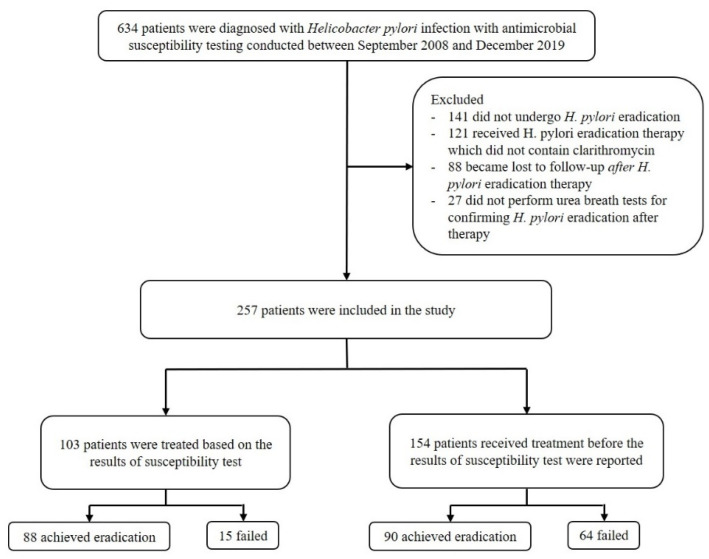
Flowchart of the process of patient enrollment.

From September 2008 to December 2019, a total of 634 patients were diagnosed with *H. pylori* infection and underwent upper gastrointestinal endoscopies to obtain gastric mucosal tissues cultured for antimicrobial susceptibility tests before the initiation of eradication therapy at the Asan Medical Center, Seoul, Korea. In total, 377 patients were excluded (141 did not undergo *H. pylori* eradication therapy, 121 received *H. pylori* eradication therapy that did not contain clarithromycin, 88 became lost to follow-up after eradication therapy, and 27 did not perform urea breath tests after completing therapy). Finally, 257 patients who received clarithromycin-containing regimens and underwent urea breath tests for confirming eradication at least four weeks after completing therapy were included in the study. They were divided into two groups: the susceptibility-guided therapy group (*n* = 103), who were treated based on the results of susceptibility testing (for example, when the strains were resistant to clarithromycin, the patient did not receive clarithromycin-containing regimen), and the empirical therapy group (*n* = 154), who were treated before the results of the susceptibility test were reported (Figure 1).

TT consisted of twice-daily amoxicillin (1000 mg), clarithromycin (500 mg), and a standard dose of proton pump inhibitor for one or two weeks. SET for 10 days consisted of twice-daily amoxicillin (1000 mg) and a standard dose of proton pump inhibitor for five days, followed by twice-daily clarithromycin (500 mg), metronidazole (500 mg), and a standard dose of proton pump inhibitor for another five days.

Ethical approval for this study was obtained from the Institutional Review Board of the Asan Medical Center. (IRB number; 2020–0518).

### 2.2. Isolation and Culture of H. pylori

The culture medium plates were Brucella broth agar that was supplemented with 5% sheep blood (Becton, Dickinson, NJ, USA), vancomycin (10 μg/mL), trimethoprim (5 μg/mL), amphotericin B (5 μg/mL), and polymyxin B (2.5 IU) (Sigma-Aldrich, Darmstadt, Germany). The plates were incubated under 37 °C in microaerophilic conditions for three to seven days (Forma Series II Water Jacketed CO_2_ Incubator Model 3131, Thermo Fisher Scientific, Waltham, MA, USA). *H. pylori* colonies were confirmed by gram staining, a positive reaction with urease, catalase, and oxidase tests, and glmM PCR. Real-time PCR was performed on an Applied Biosystems 2720 thermal cycler (Life Technologies, Carlsbad, CA, USA). All the cultured isolates were maintained at 70 °C in tryptic soy broth (Becton, Dickinson) supplemented with 15% glycerol (Sigma-Aldrich).

### 2.3. Determination of the Minimum Inhibitory Concentration (MIC)

The susceptibilities of the *H. pylori* isolates were examined by using the agar dilution method. Briefly, the bacteria were subcultured on Mueller–Hinton agar supplemented with 5% defibrinated sheep blood (Becton, Dickinson) for 48 hours. The bacterial suspension was adjusted to approximately 2.0 McFarland (1 × 107 to 1 × 108 CFU/mL) and was inoculated directly on each antibiotic-containing agar dilution plate. After incubation for 72 h, the MIC of each antibiotic was determined. The standard strain *H. pylori* ATCC 43504 was included in the susceptibility testing as a control. The resistance breakpoints for clarithromycin, amoxicillin and metronidazole were defined as ≥1.0, ≥0.5 and ≥8.0, respectively [21,22,23].

### 2.4. 13C-Urea Breath Test

The 13C-urea breath test is recommended to perform for confirmation of *H. pylori* eradication by multiple practical guidelines [6,11,18]. After fasting for at least 4 hours, a breath sample was obtained from the enrolled patients. A tablet containing 100 mg ^13^C-urea (UBIT® tablet; Otsuka Pharmaceutical Co. Ltd., Tokyo, Japan) was administered with 100 mL of water orally to patients. Then, 20 minutes after taking a tablet, a second breath sample was collected. The presence of *H. pylori* was investigated through the ^13^C-urea–urea breath test (Analyzer POCone, Otsuka Electronics Co. Ltd., Osaka, Japan) using the obtained breath samples. The cutoff value used was 2.5‰.

### 2.5. Statistical Analyses

All statistical analyses were conducted using SPSS 24.0 for Windows (SPSS Inc., Chicago, IL, USA). Categorical variables were analyzed using the chi-squared test or Fisher’s exact test. Continuous variables were analyzed using Student’s *t*-test and were reported as the mean with the standard deviation. *P*-values < 0.05 were considered as statistically significant.

## 3. Results

### 3.1. Baseline Characteristics

The baseline characteristics of the patients are shown in Table 1. There were no statistically significant differences regarding age, sex, and alcohol consumption between the two groups. The overall resistance rates to clarithromycin, amoxicillin, and metronidazole were 24.9%, 7.0%, and 34.6%, respectively. Multidrug resistance involving clarithromycin, metronidazole, and amoxicillin was 13.6% (35/257).

### 3.2. Outcomes

**Figure 2:**
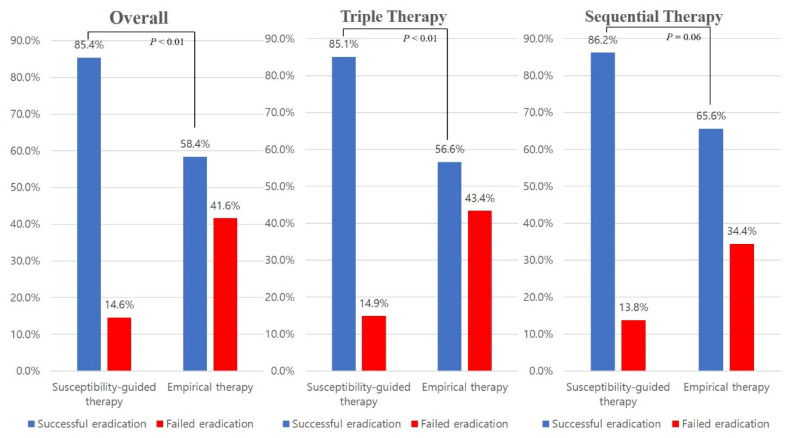
Comparison of the eradication rates between the susceptibility-guided therapy group and the empirical therapy group.

The overall eradication rate was 69.3% (178/257). In the susceptibility-guided therapy group, the eradication rate was 85.4% (88/103), while that in the empirical therapy group was 58.4% (90/154) (Table 2). There was a statistically significant difference regarding the eradication rate between the susceptibility-guided therapy and the empirical therapy groups (*P* < 0.01). TT had a significant difference between the two groups (85.1% versus 56.6%, *P* < 0.01), while SET had a moderate trend toward significance (86.2% versus 65.6%, *P* = 0.06) (Figure 2).

### 3.3. Antimicrobial Susceptibility

According to the antimicrobial susceptibility testing results, TT had an eradication rate of 84.6%, in the case of strains susceptible to both clarithromycin and amoxicillin, and 11.1% in the case of strains concurrently resistant to both these antimicrobials. SET had an eradication rate of 89.5% when the strains were susceptible concurrently to clarithromycin, amoxicillin, and metronidazole, whereas it was 0% when the strains were concurrently resistant to clarithromycin and metronidazole (Table 3). When the strains were susceptible to clarithromycin, the eradication rate was 85.0% (164/193). On the other hand, when the strains were resistant to clarithromycin, the eradication rate was 21.9% (14/64).

TT and SET had eradication rates of 83.9% and 88.0%, respectively with strains susceptible to clarithromycin (*P* = 0.49), and those of 22.6% and 18.2%, respectively with strains resistant to clarithromycin (*P* > 0.99) (Appendix A).

**Figure 3:**
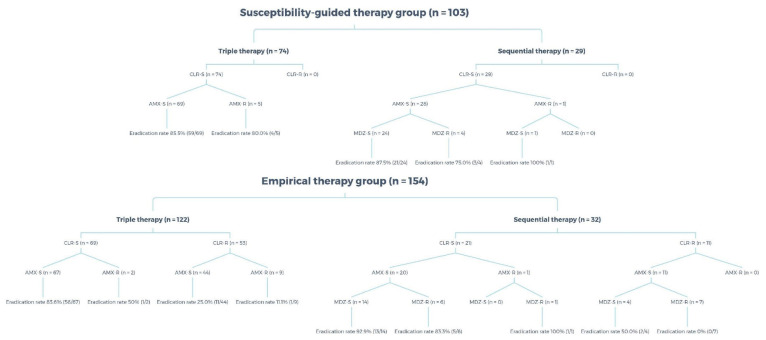
Antimicrobial susceptibility of the susceptibility-guided therapy and the empirical therapy groups. CLR, clarithromycin; AMX, amoxicillin; MDZ, metronidazole; S, susceptible; R, resistant.

Fifty-three patients were prescribed TT even though their strains were resistant to clarithromycin, with an eradication rate as low as 25.0% and 11.1%, when they were susceptible and resistant to amoxicillin, respectively. Seven patients whose strains were resistant to both clarithromycin and metronidazole were treated using SET, with an eradication rate of 0% (Table 4). Figure 3 reveals the susceptibility results of each group. In the susceptibility-guided therapy group, 74 patients received TT, and none of their strains were resistant to clarithromycin. The other 29 patients received SET, and none of their strains were resistant to clarithromycin.

### 3.4. Second-line Treatment

Among those who failed to achieve eradication (*n* = 79), 56 patients (70.9%) started second-line treatment (Table 5). Forty-eight patients received quadruple therapy, five patients were prescribed a regimen composed of a proton pump inhibitor, bismuth, metronidazole, tetracycline, and amoxicillin, and three patients received others. Quadruple therapy as second-line treatment had an eradication rate of 72.9% in the intention-to-treat analysis and 83.3% in the per-protocol analysis.

## 4. Discussion

This study aimed to compare susceptibility-guided therapy with empirical therapy as a first-line treatment containing clarithromycin for *H. pylori* eradication and to evaluate the corresponding eradication rates of these regimens according to antimicrobial susceptibility. To the best of our knowledge, this is the largest single-center and is the first study, including sequential therapy comparing susceptibility-guided therapy with empirical therapy for *H. pylori* eradication. The obtained results documented that the eradication rate of susceptibility-guided therapy was 85.4% and that of empirical therapy was 58.4%, which showed a significant difference (*P* < 0.01).

TT is used widely as a first-line treatment for *H. pylori* eradication because of the simplicity and cost benefits associated with this regimen. SET was first introduced by Zullo et al. in 2000 as a new regimen for *H. pylori* eradication [24]. The efficacy of these two regimens, however, has decreased due to increased antibiotic resistance, particularly to clarithromycin [14,15,16,17]. The eradication rate with TT varied between the susceptibility-guided therapy and the empirical therapy groups, at 85.1% and 56.6%, respectively (*P* < 0.01). The eradication rate with SET in the susceptibility-guided therapy group was higher than that in the empirical therapy group with a moderate trend toward significance (86.2% versus 65.6%, *P* = 0.06). Moreover, when *H. pylori* was susceptible to both clarithromycin and amoxicillin, TT showed an eradication rate of 84.6%. SET showed an eradication rate of 89.5% when the strains were susceptible to clarithromycin, amoxicillin, and metronidazole, while it was 0% in strains concurrently resistant to clarithromycin and metronidazole. The eradication rate with TT was 71.4% when the strains were susceptible to clarithromycin but resistant to amoxicillin. However, when the strains were resistant to clarithromycin, the eradication rate with TT was as low as 25.0% (susceptible to amoxicillin) and 11.1% (resistant to amoxicillin). In addition, the eradication rate with SET was 50.0% when the strains were resistant to clarithromycin but susceptible to metronidazole. On the other hand, when the strains were resistant to both clarithromycin and metronidazole, the eradication rate with SET was 0%. It was suggested that the eradication rate with TT is influenced mostly by clarithromycin resistance but that SET is affected not only by clarithromycin resistance but also by metronidazole resistance. Other studies indicated that clarithromycin resistance has a lesser influence on the efficacy of SET than that of TT [25,26].

The resistance rate to clarithromycin, amoxicillin, and metronidazole in this study was 24.9%, 7.0% and 34.6%, respectively, which were in accordance with the resistance rates in Korea as reported previously [27,28]. Thirty-five patients showed multidrug resistance involving those three antibiotics (35/257, 13.6%), which was in accordance with the previously reported data in Korea as 11.2% [28]. There is no treatment consensus for multidrug-resistant *H. pylori* treatment, and antimicrobial susceptibility testing is increasingly important [29]. In the empirical therapy group, the clarithromycin resistance rate was 41.6%. This high resistance rate could be explained by the increasing resistance rates in Korea, from 43.7% in 2015–2016 to 45.9% in 2017–2018, as reported by Lee et al. [27], because in this study, most patients of the empirical therapy group underwent antimicrobial susceptibility tests from 2015–2018 (135 of 154). In addition, this study revealed that the eradication rates with TT and SET were 67.3% and 75.4%, respectively, similar to previously assumed eradication rates [30].

When the strains were susceptible to both clarithromycin and amoxicillin, TT showed an eradication rate of 85.5% in the susceptibility-guided therapy group and 83.6% in the empirical therapy group, both of which were lower than 90%. Lower-than-expected eradication rates could be explained by the patients’ poor compliance with the treatment [31,32] and antimicrobial heteroresistance, defined as the presence of heterogeneous populations of co-existing bacteria that have different levels of antibiotic resistance from one another [33]. Lee et al. reported that approximately 10% of patients failed to take eradication therapy medications [34]. Unfortunately, due to a lack of medical records, we could not investigate the treatment compliance of the participants in this study. Matteo et al. documented that two *H. pylori* strain obtained from one person’s gastric mucosa significantly differed in antimicrobial susceptibility [35]. In this study, however, we could not investigate the biopsy sites and the number of obtained biopsies due to a lack of medical records. Moreover, in TT, a low proportion of 14-day treatment duration rather than 7-day treatment duration may explain the lower eradication rate. Among those, who received TT in the susceptibility-guided therapy group, the 14-day treatment regimen was administered only to 10.8% of the patients (8/66), whose eradication rate was 100%. However, the eradication rates were not statistically significant differences between the 7- and 14-day treatment duration. Similarly, strains susceptible to all the three antibiotics had an eradication rate with SET at 87.5% and 92.9% in the susceptibility-guided therapy and the empirical therapy groups, respectively, which were not 100%.

Among the 56 patients, who started second-line treatment after failing the first-line treatment, 49 received quadruple therapy, as suggested by multiple practical guidelines [6,11,18]. The results showed that the eradication rate with second-line quadruple therapy was 83.3%, which was in accordance with the nationwide *H. pylori* registry study in Korea [36]. After the failure of second-line treatment, there is no treatment consensus. Levofloxacin triple regimen or rifabutin triple regimen is suggested as rescue therapy by the guidelines [6,18]. However, Lim et al. reported in 2017 that the eradication rate of levofloxacin triple regimen as third-line treatment was 56.9% in Korea [37]. This low eradication rate can be explained by the high-resistance rate to levofloxacin in Korea [28,38]. Rifabutin triple regimen has several limitations. First, this drug is expensive. Second, myelotoxicity causing severe leukopenia or thrombocytopenia has been reported. Finally, there is an opinion that the use of rifabutin should be reserved for *Mycobacterium tuberculosis* because multidrug-resistant *M. tuberculosis* has increased [39]. More efforts and studies regarding rescue therapy after the failure of second-line therapy are required.

From this study, amoxicillin was found to play a less decisive role in TT. The eradication rate of the amoxicillin-susceptible strains with TT was not much different from that of amoxicillin-resistant strains when the strains were clarithromycin-susceptible (84.6% vs. 71.4%, *P* = 0.36). Metronidazole was also found to play an important role in SET. The eradication rate of SET was 0% when the strains were resistant to both clarithromycin and metronidazole in the empirical therapy group.

There are several limitations to this study. First, this is a retrospective, observational, single-center study, so it may limit generalizing the results. Second, the compliance of the participants and the adverse reactions related to *H. pylori* eradication therapy were not investigated. Compliance with therapy is accepted as the single most important factor in *H. pylori* eradication, while a major cause of poor compliance is the presence of adverse reactions. Third, the biopsy sites and the number of obtained biopsies were not investigated. Thus, antimicrobial heteroresistance, which is a risk factor for lower-than-expected eradication rates, was not examined. Lastly, empirical therapy was performed more frequently despite susceptibility testing being carried out. Failure of *H. pylori* culture is not common [19,20], and it takes more than 10 days, sometimes as long as two weeks, to obtain the results of susceptibility testing [21]. More than half of the participants came to the outpatient clinics before the susceptibility testing results were reported, and a majority of them wanted to start *H. pylori* eradication therapy as soon as possible. Because multiple practical guidelines recommend empirical therapy as first-line treatment for *H. pylori*, there were no medical issues regarding the provision of this regimen.

In conclusion, susceptibility-guided therapy based on antimicrobial susceptibility tests as clarithromycin-containing first-line treatment for *H. pylori* improved the eradication rate of TT and SET by 28.5 (*P* < 0.01) and 20.6 (*P* = 0.06) percent points, respectively, compared with empirical therapy.

## Figures and Tables

**Table 1:** Clinical characteristics.

	TOTAL	Susceptibility-Guided Therapy	Empirical Therapy	*P*-Value
Number	257	103	154	
Age (mean ± SD)	58.3 ± 10.6	57.6 ± 10.5	58.8 ± 10.7	0.41
Male (%)	147 (57.2%)	59 (57.3%)	88 (57.1%)	0.98
Smoking (%)	41 (16.0%)	17 (16.5%)	24 (15.6%)	0.86
Alcohol (%)	126 (49.0%)	49 (47.6%)	77 (50.0%)	0.80
Indications				0.34
Early gastric cancer (%)	166 (64.6%)	69 (67.0%)	97 (63.0%)	
Atrophic gastritis (%)	24 (9.3%)	10 (9.7%)	14 (9.1%)	
MALT lymphoma (%)	24 (9.3%)	7 (6.8%)	17 (11.0%)	
Gastric adenoma (%)	19 (7.4%)	7 (6.8%)	12 (7.8%)	
Peptic ulcer (%)	6 (2.3%)	0 (0%)	6 (3.9%)	
Functional dyspepsia (%)	6 (2.3%)	4 (3.9%)	2 (1.3%)	
Others (%)	12 (4.7%)	5 (4.9%)	6 (3.9%)	
Resistance				
CLR-R (%)	63 (24.9%)	0 (0%)	64 (41.6%)	<0.01
AMX-R (%)	18 (7.0%)	6 (5.8%)	12 (7.8%)	0.55
MDZ-R (%)	89 (34.6%)	31 (30.1%)	58 (37.7%)	0.21

SD, standard deviation; MALT, mucosa-associated lymphoid tissue; CLR, clarithromycin; AMX, amoxicillin; MDZ, metronidazole; R, resistant.

**Table 2:** Eradication rates comparing susceptibility-guided therapy with empirical therapy.

Eradication	TOTAL	Susceptibility-Guided Therapy	Empirical Therapy	*P*-Value
Overall (*n* = 257)		103	154	
Success (%)	178 (69.3%)	88 (85.4%)	90 (58.4%)	<0.01
Failure (%)	79 (30.7%)	15 (14.6%)	64 (41.6%)	
TT (*n* = 196)		74	122	
Success (%)	132 (67.3%)	63 (85.1%)	69 (56.6%)	<0.01
Failure (%)	64 (32.7%)	11 (14.9%)	53 (43.4%)	
SET (*n* = 61)		29	32	
Success (%)	46 (75.4%)	25 (86.2%)	21 (65.6%)	0.06
Failure (%)	15 (24.6%)	4 (13.8%)	11 (34.4%)	

TT = triple therapy; SET = sequential therapy.

**Table 3:** Antimicrobial susceptibility testing results.

Antimicrobial Susceptibility Testing in TT				Number (%) of Successful Eradication
Clarithromycin	Amoxicillin			
S	S			115/136 (84.6)
S	R			5/7 (71.4)
R	S			11/44 (25.0)
R	R			1/9 (11.1)
**Antimicrobial susceptibility testing in SET**				Number (%) of successful eradication
Clarithromycin	Amoxicillin	Metronidazole		
S	S	S		34/38 (89.5)
S	S	R		8/10 (80.0)
S	R	S		1/1 (100)
S	R	R		1/1 (100)
R	S	S		2/4 (50.0)
R	S	R		0/7 (0)

S, susceptible; R, resistant.

**Table 4:** Antimicrobial susceptibility testing results in the susceptibility and empirical therapy groups.

Antimicrobial Susceptibility	Eradication Rate
Susceptibility-guided therapy group	*n* = 103
Triple therapy	*n* = 74
CLR-S and AMX-S	85.5% (59/69)
CLR-S and AMX-R	80.0% (4/5)
Sequential therapy	*n* = 29
CLR-S and AMX-S and MDZ-S	87.5% (21/24)
CLR-S and AMX-S and MDZ-R	75.0% (3/4)
CLR-S and AMX-R and MDZ-S	100.0% (1/1)
Empirical therapy group	*n* = 154
Triple therapy	*n* = 122
CLR-S and AMX-S	83.6% (56/67)
CLR-S and AMX-R	50.0% (1/2)
CLR-R and AMX-S	25.0% (11/44)
CLR-R and AMX-R	11.1% (1/9)
Sequential therapy	*n* = 32
CLR-S and AMX-S and MDZ-S	92.9% (13/14)
CLR-S and AMX-S and MDZ-R	83.3% (5/6)
CLR-S and AMX-R and MDZ-R	100% (1/1)
CLR-R and AMX-S and MDZ-S	50.0% (2/4)
CLR-R and AMX-S and MDZ-R	0% (0/7)

CLR, clarithromycin; AMX, amoxicillin; MDZ, metronidazole; S, susceptible; R, resistant.

**Table 5:** Second-line treatment.

Treatment	Success	Failure	Loss to Follow-Up
Total (*n* = 56)	40 (71.4%)	9 (16.1%)	7 (12.5%)
Quadruple (*n* = 48)	35 (72.9%)	7 (14.6%)	6 (12.5%)
PBAMT (*n* = 5)	4 (80.0%)	1 (20.0%)	0
Others (*n* = 3)	1 (33.3%)	2 (66.7%)	0

PBAMT, proton pump inhibitor, bismuth, metronidazole, tetracycline, and amoxicillin.

**Figure A1:**
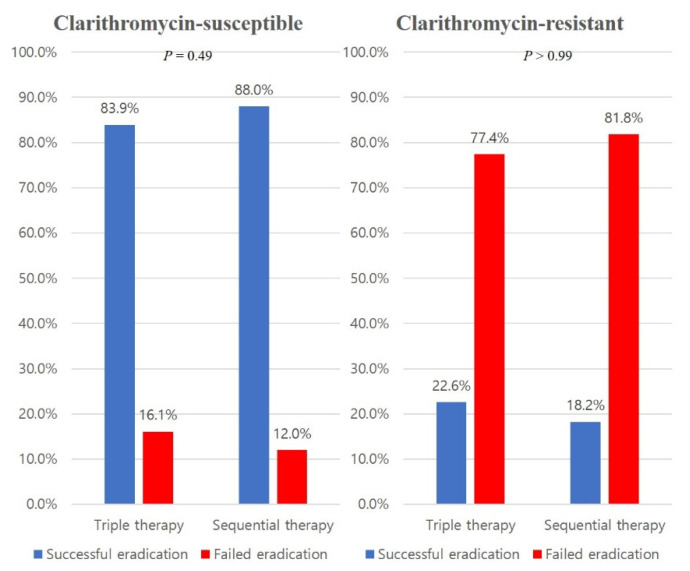
Comparison of the eradication rates between triple therapy and sequential therapy according to clarithromycin susceptibility.

## Data Availability

The data presented in this study are available on request from the corresponding author. The data are not publicly available due to privacy.
